# Protective effects of pomegranate seed oil on ovariectomized rats as a model of postmenopausal osteoporosis: A multi-detector computed tomography evaluation

**Published:** 2014

**Authors:** Morteza Saravani, Hossein Kazemi Mehrjerdi, Ali Mirshahi, Amir Afkhami Goli

**Affiliations:** 1 *DVM Graduated student of Faculty of Veterinary Medicine, Ferdowsi University of Mashhad, Mashhad, Iran; *; 2 * Department of Clinical Sciences, Faculty of Veterinary Medicine, Ferdowsi University of Mashhad, Mashhad, Iran; *; 3 * Department of Basic Sciences, Faculty of Veterinary Medicine, Ferdowsi University of Mashhad, Mashhad, Iran.*

**Keywords:** Computed Tomography, Osteoporosis, Pomegranate seed oil, Rat, Vertebral column

## Abstract

The pomegranate seed oil (PSO), containing 17-α-estradiol, is one of the newly found phytosterols with synergistic health effects on estrogen related physiological conditions. Herein, PSO was assessed for its potential improving effects on bone characteristics in a rat model of menopausal syndrome. Three month old non-pregnant female Wistar rats (n = 30) were either sham-operated (SHAM) or ovariectomized (OVX), each divided into two further groups receiving 0.1 mL PSO or the same volume of paraffin oil as placebo. Before the operation and 67 days after it, multi-detector computed tomography (MDCT) scanning was performed with the identical setup option for the scanner to measure the bone mineral density (BMD) in body of 12^th^ thoracic vertebra, 1^st^ to 6^th^ lumbar vertebra and sacrum. This study revealed that bone density of 1^st^, 3^rd^, 5^th^, 6^th^ and sacrum body were significantly different between OVX and SHAM groups during the study period. In conclusion, PSO during 67 days study could not completely prevent the osteoporotic effects caused by ovariectomy in vertebral column of rats.

## Introduction

Osteoporosis is one of the most frequent diseases affecting one in ten people in the world and one in three women in their fifties. It is a systemic, chronic and metabolic bone disease characterized by low bone mass and a micro-architectural deterioration of bone tissue leading to bone fragility and an increase in bone fracture. Low bone mass results from genetic, nutritional and lifestyle factors, the use of drugs and decreased estrogen levels.^1^ Estrogen deficiency is responsible for bone loss in postmenopausal women.^2^ Involutional bone loss in postmenopausal women has been suggested to occur in the two phases: an early rapid phase beginning at menopause and lasting for 6 to 10 years; and a subsequent slow phase lasting for the rest of a woman’s life. It is also known that estrogen replacement therapy can prevent the early phase of involutional bone loss and also restore the rate of bone resorption and formation to premenopausal levels in menopausal women. Phytoestrogens are estrogen agonists and can be effective especially when presence of estrogen is decreased.^3^

Pomegranate (*Punica granatum*) is a native plant from Iran. One of the most remarkable characteristics of pomegranate fruit is that its seeds are rich with estrogens. Pomegranate seeds are known to contain the estrogenic compounds, estrone and estradiol that are chemically identical to those biosynthesized in human body.^3^ Since pomegranate seed oil (PSO) contains phytoestrogens, this prediction may be true that pomegranate seed oil is able to prevent bone loss in postmenopausal women.

Rats are currently principal laboratory animals used to investigate this disease, since they are inexpensive to maintain, grow rapidly, have a relatively short lifespan and are widely available. There are several methods inducing postmenopausal osteoporosis in rats including hypophysectomy, parathyroidectomy and ovariectomy. The latter one is considered to give reliable model of post-menopausal osteoporosis.^4^

Osteoporosis can be diagnosed and assessed quantitatively by measuring bone mineral density (BMD). Multi-detector computed tomography (MDCT) is one of the most accurate and precise absorptiometry methods for evaluation of BMD. Compared with conventional computed tomography (CT), MDCT offers increased spatial resolution, isotropic imaging and faster data acquisition. Although peripheral quantitative computed tomography (pQCT) is the main method of BMD measurement in small animals, MDCT is much more widely available. Besides, examination time per animal is much shorter for MDCT.^5^

The aim of this study was to find whether PSO can prevent bone loss and whether MDCT could precisely and accurately predict bone loss in ovariectomized rat model of postmenopausal osteoporosis or not.

## Materials and Methods

Three month old female non pregnant Wistar rats, weighing 175 to 200 g (n = 30) were used as the experimental animals in this study. The animals were housed in special plastic rat cages with wood shavings in environmentally controlled laboratory upon arrival and acclimatized for seven days. They were fed with standard laboratory animal food and allowed water *ad libitum. *Then, the animals were chosen randomly and were either ovariectomized (OVX) or sham operated (SHAM) and divided into four groups: OVX + PSO (n = 8), OVX (n = 8), SHAM + PSO (n = 7) and SHAM (n = 7). 

The PSO used in this study was produced, blended and generously donated by Orum Narin^®^ company (Urmia, Iran) and confirmed to contain estrogens (1.3 µg mL^-1^) by immune-radiometric assay (IRMA) using commercial kits (Biosource, Dorest, Belgium) and a Dream Gamma- 5 gamma counter (Shin Jin Medics Inc., Korea); the intra-assay CVs in all IRMA runs were less than 3.4%. Considering the importance of estrogen levels in our model we have chosen the amount of PSO based on the measured estrogen levels in PSO, and the normal levels of estrogen in female rats, as we aimed to use the estrogen containing PSO as a substitute for normal decreased estrogen in ovariectomized rats. The rats were gavaged with PSO (0.1 mg) every day for 67 days starting on the next day of ovariectomy. To SHAM and OVX groups, paraffin oil was administrated similarly (0.1 mg) as placebo.

To assess bone loss due to OVX and treatment, rats were anesthetized and scanned before surgery (baseline) and at the end of treatment (day 67 after surgery) using MDCT equipped with appropriate software (MaxViewer^®^; Neusoft, NeuViz 16, Shenyang, China) for BMD assessment in small laboratory animals. For each animal the density of body of 12^th^ thoracic vertebra, 1^st^ to 6^th^ lumbar vertebra and sacrum was evaluated according to Hounsfield unit (HU).^5^ For the same vertebral body in different rats, multiple slice acquisition with 0.75 mm slice thickness was used (**Fig. 1**).

Mean ± SD of vertebrae density at day 0 and 67 were calculated. Repeated measures ANOVA followed by Tukey‘s post-hoc were conducted to investigate the difference between groups during the study period. A *p*-values less than 0.05 were considered as significant. All statistical analyses were performed using SPSS statistical software (Version 16; SPSS Inc., Chicago, USA).

## Results

Descriptive statistics, indicating the mean and standard deviation (SD) of body density of vertebrates for different groups are presented in Table 1.

In the SHAM+PSO group, mean value for bone density of 1^st^ lumbar vertebra was significantly higher than OVX+ PSO and OVX groups during the study period (*p* < 0.05). Also, in SHAM group, this parameter was significantly higher than OVX+PSO and OVX groups (*p* < 0.05).

Mean value for body density of 3^rd^ lumbar vertebra for SHAM+PSO group was significantly higher than OVX+PSO group (*p* < 0.05). 

When body density was measured at the 5^th^ lumbar vertebra, the SHAM+PSO and SHAM groups had greater mean value than OVX+PSO group (*p* < 0.05). 

Mean value for body density of 6^th^ lumbar vertebra for SHAM+PSO group was significantly higher than OVX+PSO group and OVX group (*p* < 0.05). Additionally in SHAM group, this parameter was also significantly higher than OVX+PSO group and OVX group (*p* < 0.05). 

Animals in the OVX+PSO group had significantly lower body density of the sacrum bone compared with the SHAM+PSO group (*p* < 0.05). 

Body density of 12^th^ thoracic vertebra, 2^nd^ and 4^th^ lumbar vertebra was not different between groups during the study period (*p* > 0.05).

**Table 1:** Mean and standard deviation (SD) of body density of vertebrates on day 0 and 67 for different groups according to Hounsfield unit

**Vertebra**	**Group** *	**No.**	**Baseline**		**Day 67**	
			**Mean**	**SD**	**Mean**	**SD**
**12** ^th^ ** thoracic **	OVX+PSO	8	570.83	84.97	705.78	98.39
	OVX	8	613.50	33.34	698.54	95.17
	SHAM+PSO	7	599.63	63.14	771.16	61.90
	SHAM	7	655.72	43.37	722.66	29.75
**1** ^st^ ** lumbar **	OVX+PSO^a^	8	568.00	62.85	579.46	67.14
	OVX^a^	8	612.80	32.59	530.02	59.90
	SHAM+PSO^b^	7	636.91	36.87	702.88	58.06
	SHAM^b^	7	644.72	32.89	716.30	63.79
**2** ^nd^ ** lumbar **	OVX+PSO	8	593.25	66.38	521.03	62.13
	OVX	8	627.66	36.70	538.60	59.18
	SHAM+PSO	7	604.00	82.87	696.85	74.50
	SHAM	7	644.72	76.35	629.12	67.42
**3** ^rd^ ** lumbar **	OVX+PSO^a^	8	574.50	67.11	531.10	67.39
	OVX^ab^	8	626.80	47.91	540.75	78.16
	SHAM+PSO^b^	7	614.66	87.20	675.34	70.42
	SHAM^ab^	7	633.25	83.57	620.98	14.22
**4** ^th^ ** lumbar **	OVX+PSO	8	588.91	83.32	565.05	73.61
	OVX	8	632.88	50.16	581.18	73.37
	SHAM+PSO	7	635.44	84.58	667.28	80.25
	SHAM	7	640.52	105.65	712.29	69.59
**5** ^th^ ** lumbar **	OVX+PSO^a^	8	583.93	77.12	582.73	80.72
	OVX^ab^	8	666.44	51.05	558.88	71.86
	SHAM+PSO^b^	7	661.90	68.90	730.55	73.92
	SHAM^b^	7	678.97	73.64	678.30	37.30
**6** ^th^ ** lumbar **	OVX+PSO^a^	8	634.63	67.04	589.35	66.43
	OVX^a^	8	690.73	46.57	545.53	79.47
	SHAM+PSO^b^	7	670.63	55.74	740.89	92.36
	SHAM^b^	7	720.75	64.47	700.63	63.19
**Sacrum**	OVX+PSO^a^	8	661.38	88.46	632.77	91.71
	OVX^ab^	8	706.03	75.26	611.92	71.53
	SHAM+PSO^b^	7	717.65	80.71	788.69	77.09
	SHAM^ab^	7	752.60	65.84	689.27	26.14

* OVX: Ovariectomized; PSO: Pomegranate seed oil; SHAM: Sham operated.

ab For each vertebra, groups which are followed by different superscripts are significantly different (*p *< 0.05).

## Discussion

Menopause results in elevated bone turnover, an imbalance between bone formation and bone resorption and net bone loss.^6^ Estrogen replacement therapy is commonly used to cure this disease in postmenopausal women. However, its effect on bone mineral density depends greatly on the time the therapy is initiated, and its total duration.^7^

Many animal models were used in osteoporosis research but among them rodents have numerous advantages, as they are inexpensive, easy to house, have a rapid generation time, and their skeletons are sensitive to the loss of ovarian hormones. The ovariectomized rat model is most commonly used in research on post-menopausal osteoporosis.^8^

Many studies have demonstrated bone loss induced by estrogen deficiency in ovariectomized rats. Lei *et al.* reported a significant decrease in femoral BMD in 8-week OVX rats as compared with SHAM rats.^9^ In a recent study of histological observations of bone resections of vertebrae and femurs, bone mass was remarkably reduced in ovariectomized rats than in sham group.^10^

The PSO treatment had been reported earlier for its beneficial effects on osteoporosis in rats. In 2004, Mori-Okamoto *et al*. investigated the efficiency of PSO treatment on ovariectomized rat as a model of postmenopausal osteoporosis. Their result showed that BMD in OVX+PSO group of rats are higher than OVX group and lower than SHAM group. They concluded that PSO is able to prevent bone loss in OVX rats.^3^

Ferreti *et al.* showed that osteoporotic changes could be detectable in lumbar vertebrae of rats using histomorphometry 60 days after ovariectomy.^12^ In the present study, we investigated changes in BMD and bone loss after ovariectomy in Wistar rats using MDCT. At 67 days after ovariectomy, density of 1^st^, 3^rd^, 5^th^ and 6^th^ lumbar vertebra and sacrum body in the OVX groups (OVX and OVX+PSO) are significantly lower than those of in the SHAM groups (SHAM and SHAM+PSO). These findings support the notion that our animal model successfully mimicked the effects of osteoporosis caused by the lack of ovarian hormone in the OVX model but does not support the efficiency of PSO in prevention and treatment of post-menopausal osteoporosis. 

Peripheral quantitative computed tomography is the main method of bone mineral density measurement in small animals. However, pQCT is usually only available in specialized centers, while MDCT is much more widely available. Scanned MDCT images can separate trabecular and cortical bone, and therefore, they can be used to produce realistic depiction of apparent and tissue BMDs, which in turn reflect degrees of bone mineralization.^5^ In 2009, for the first time Wang *et al.* used MDCT for lumbar vertebrae bone densitometry in rats. They found this method as accurate and precise as pQCT, though MDCT lumbar spine densitometry was much quicker than pQCT densitometry, taking 5 min rather than 30 min to complete. The reduced examination time of MDCT densitometry should also help minimize animal stress from anesthesia. Faster acquisition time afforded by MDCT also allows other parts of the skeleton, such as the thoracic spine, pelvis and femora, to be included in the scan plane, while isotropic reconstruction should allow easier vertebral body numbering, easier detection of vertebral anomalies that could influence BMD, and better assessment of rat skeleton morphology. Another potential advantage of MDCT over pQCT is that the larger gantry size should allow two to four animal cradles to be to be aligned side by side, enabling simultaneous imaging of more than one animal.^5^

When considering animal models of osteoporosis, it is important that bone sites should be taken into account. It is well known that, in ovariectomized animals, as in postmenopausal women, bone loss induced by ovarian deficiency mainly results from trabecular bone loss.^11^ In a study of ovariectomized rats, Ferreti *et al.* reported that bone mass started to decrease earlier and more extensively in trabecular than in cortical bone.^12^ Therefore trabecular bones are early detectors of bone loss in animal models of postmenopausal osteoporosis. 

In conclusion, the present study demonstrates that the ovariectomized rat model offers a reliable and reproducible model of osteoporosis. Furthermore, it shows a significant loss in trabecular bone in lumbar vertebrae almost 10 weeks after ovariectomy. Finally our study does not support the potential efficacy of PSO treatment in prevention and treatment of ovariectomy-induced osteoporosis on lumbar vertebrae and sacrum bone in rat.
